# The human microbiome as a reservoir of antimicrobial resistance

**DOI:** 10.3389/fmicb.2013.00087

**Published:** 2013-04-17

**Authors:** John Penders, Ellen E. Stobberingh, Paul H. M. Savelkoul, Petra F. G. Wolffs

**Affiliations:** Department of Medical Microbiology, Maastricht University Medical Centre+Maastricht, Netherlands

**Keywords:** antimicrobial resistance, resistome, metagenomics, gut microbiota, microbiome

## Abstract

The gut microbiota is amongst the most densely populated microbial ecosystem on earth. While the microbiome exerts numerous health beneficial functions, the high density of micro-organisms within this ecosystem also facilitates horizontal transfer of antimicrobial resistance (AMR) genes to potential pathogenic bacteria. Over the past decades antibiotic susceptibility testing of specific indicator bacteria from the microbiome, such as *Escherichia coli*, has been the method of choice in most studies. These studies have greatly enlarged our understanding on the prevalence and distribution of AMR and associated risk factors. Recent studies using (functional) metagenomics, however, highlighted the unappreciated diversity of AMR genes in the human microbiome and identified genes that had not been described previously. Next to metagenomics, more targeted approaches such as polymerase chain reaction for detection and quantification of AMR genes within a population are promising, in particular for large-scale epidemiological screening. Here we present an overview of the indigenous microbiota as a reservoir of AMR genes, the current knowledge on this “resistome” and the recent and upcoming advances in the molecular diagnostic approaches to unravel this reservoir.

## INTRODUCTION

Antimicrobial resistance (AMR) is worldwide one of the most important public health threats that we face currently. AMR reduces clinical efficacy and increases treatment costs. Furthermore, AMR jeopardizes the achievements of modern medicine, since the success of interventions such as organ transplantation, cancer chemotherapy, and major surgery depends on effective antimicrobial agents for prevention and treatment of (nosocomial) infections. With a lack of novel antibiotics in the pipeline, the conservation of existing ones is crucial.

While the majority of studies on the epidemiology of antibiotic susceptibility have been focused on clinical isolates, the human microbiota warrants special attention as perhaps the most accessible reservoir of resistance genes due to the high likelihood of contact and genetic exchange with potential pathogens. Here we present a non-comprehensive overview of the indigenous microbiota as a reservoir of AMR genes, the current knowledge on this “resistome” and the recent and upcoming advances in the molecular diagnostic approaches to unravel this reservoir.

## THE HUMAN GUT MICROBIOTA

The human gut microbiota has long been recognized to contribute to health and disease by influencing gut maturation, host nutrition and pathogen resistance (Dethlefsen et al., 2006). More recently, intestinal microbes have been shown to influence host energy metabolism (Backhed et al., 2004), intestinal epithelial proliferation (Rakoff-Nahoum et al., 2004) and immune responses (Noverr and Huffnagle, 2004). As such, perturbations in the microbiota composition have been linked to vaginosis (Ravel et al., 2011), obesity (Ley et al., 2005), inflammatory bowel disease (IBD; Neuman and Nanau, 2012), functional bowel disorders (Simren et al., 2013), allergies (Penders et al., 2007), and other diseases (Kinross et al., 2011).

The establishment of the indigenous microbiota starts as soon as the amniotic membranes rupture and subsequently involves a succession of bacterial populations waxing and waning as the diet changes and the host develops.

The bacteria colonizing the infant gut during the first days of life originate mainly from the mother’s birth canal, the living environment and from handling by other individuals. (Reid et al., 2011). Vaginally born infants are colonized at first by maternal fecal and vaginal bacteria, whereas infants born through cesarean section are exposed initially to bacteria originating from the hospital environment and healthcare workers (Penders et al., 2006; Dominguez-Bello et al., 2010).

Other factors that can influence the neonatal intestinal microbiota composition are: the environment during birth, prematurity, hygiene measures, and the type of infant feeding (Penders et al., 2005, 2006; Adlerberth et al., 2007; Azad et al., 2013). The newborn’s gastrointestinal communities still have relatively few species and lineages, but diversity increases rapidly over the first year of life (Palmer et al., 2007).

The majority of indigenous microbes cannot be cultivated, and it’s only since the recent application of sequencing techniques that the human microbiota has been described in all complexity including the unculturable and rare taxa. Eckburg et al. (2005) undertook a large comparative analysis of 16S rRNA gene sequences, by means of conventional cloning and sequencing, to characterize the mucosal and fecal microbial communities in three healthy individuals. Results demonstrated that bacterial diversity within the human colon and feces was far greater than previously described, most of it being novel and uncultivated (62 and 80% of identified phylotypes, respectively). Subsequent studies using next-generation sequencing or micro-arrays targeting the 16S rRNA gene provided an even more in-depth picture of our inner microbial ecosystem. These studies amongst others demonstrated that the gut microbiota is dominated by the phyla Bacteroidetes and Firmicutes and to a lesser extent Actinobacteria, Proteobacteria, Verrucomicrobiae, and Fusobacteria (Dethlefsen et al., 2006; Andersson et al., 2008; Zoetendal et al., 2008).

Although the individual microbial composition has an “individual core” that varies on the phylotype level (Jalanka-Tuovinen et al., 2011), the overall phylogenetic profile can be categorized into three predominant variants, or “enterotypes,” dominated by *Bacteroides*, *Prevotella*, and *Ruminococcus*, respectively (Arumugam et al., 2011). In a recent study among 98 individuals, enterotypes were found to be strongly associated with long-term diets (Wu et al., 2011).

A broad gene catalog of the human gut microbiome derived from 124 adult subjects, generated by the international metagenomics of human intestinal tract (Meta-HIT) project, identified a staggering number of some 3.3 million different bacterial genes among the studied individuals, which is 150-fold more than in our own human genome. (Qin et al., 2010).

Next to the apparent beneficial effects on human health, the human microbiota warrants special attention as perhaps the most accessible reservoir of antibiotic resistance given the high density of micro-organisms within this ecosystem. The gastrointestinal tract is an open system, which every day encounters a myriad of bacterial acquisitions originating from the environment (e.g., from food, water, soil, and other humans or animals; Baquero, 2012). These incoming bacteria often harbor antibiotic resistance genes. In case of opportunistic pathogens of environmental or food-borne origin, such AMR bacteria can pose a direct threat to the host. Alternatively, these incoming microbes might transfer their resistance elements through horizontal gene transfer (HGT) to the indigenous microbial communities. HGT can occur between different species and genera, and as such between commensals and (opportunistic) pathogens. Well-known examples are the CTX-M extended-spectrum beta-lactamase (ESBL) resistance genes, which originate from Kluyvera species (Canton and Coque, 2006) or the wide distribution of typeA streptogramin acetyltransferases across bacterial species (Seoane and Garcia Lobo, 2000).

Therefore, it is crucial to study the AMR potential of natural environments, such as the indigenous microbiota, and not merely as mechanisms already emerged in pathogens (Wright, 2010; Schmieder and Edwards, 2012).

## ANTIMICROBIAL RESISTANCE IN INDICATOR MICRO-ORGANISMS.

Until very recently, costs and limitations in the advancement of molecular technology hampered the assessment of the AMR of the microbiome as a whole. Instead, the antibiotic susceptibility of indicator micro-organisms was assessed. Mostly, the choice of the indicator micro-organism(s) was based on the clinical relevance of these organisms; i.e., whether they were implicated in causing infections, but also on the cultivability of these organisms rather than for example those who represented the majority of the microbiome. Cultivability has been of major importance because even today, every method to assess antibiotic susceptibility (rather than antibiotic resistance) involves culturing the micro-organism (Okeke et al., 2011).

### CULTURE-BASED ANALYSIS OF ANTIMICROBIAL SUSCEPTIBILITY

By far the greatest majority of studies to date on antimicrobial susceptibility (AMS) of the gut microbiota have used (selective) culturing and isolation of specific micro-organisms, followed by culture-based antibiotic susceptibility testing to obtain their results. Most of these studies focused on *E. coli* or enterococci as indicator micro-organisms and they have gained invaluable insights regarding: (i) geographic differences in AMS prevalence and changes in prevalence over time (Nys et al., 2004); (ii) the influence of population density and hospitalization (Jonkers et al., 2002; Bruinsma et al., 2003a,b); and. (iii) the link between AMS in food animals and humans (van den Bogaard and Stobberingh, 2000). Additionally, culture-based analysis of AMS of indicator micro-organisms have demonstrated the relationship between the AMS of fecal *E. coli* and that of *E. coli* implicated in infections such as urinary tract infections (Lester et al., 1990; den Heijer et al., 2012). Finally, culture-based studies have significantly increased our knowledge on the relationship between antibiotic use and AMS of intestinal micro-organisms. It has been shown that antibiotic use is strongly correlated with the prevalence of resistant fecal *E. coli* and other Gram-negatives (Murray et al., 1982; Bruinsma et al., 2003c; van der Veen et al., 2009; and reviewed in Donskey, 2006), but also that resistance is emerging in populations with minimal exposure to antibiotics such as in remote Indian communities (Bartoloni et al., 2004; Grenet et al., 2004). These latter findings implicated the role of transfer of resistant bacteria from person-to-person or via food or the environment. To further study the spreading of resistance through microbial communities or from one person’s microbiota to another, molecular diagnostic analysis was introduced.

### MOLECULAR DIAGNOSTICS OF ANTIMICROBIAL RESISTANCE

In a follow-up study on the samples from a remote Indian community with minimal antibiotic exposure, Pallecchi et al. (2007) used molecular genotyping as well as the characterisation of resistance genes by polymerase chain reaction (PCR). This study supported the hypothesis that emergence of resistance within this community was due to the introduction of resistant strains from antibiotic-exposed settings, followed by the local dissemination and maintenance of resistance. The analysis of resistance genes by PCR and/or sequencing has also been extensively used to monitor the spreading of ESBL-producing Enterobacteriaceae. Several studies monitored changes in carriage of ESBL-producing micro-organisms within different geographic areas and over time (Overdevest et al., 2011; Geser et al., 2012; Gijon et al., 2012; Severin et al., 2012). PCR and sequencing analysis have also shed light on the vehicles of transmission of resistance. The role of resistance-carrying integrons was identified after antibiotic treatment (Vinue et al., 2008; van der Veen et al., 2009; Vo et al., 2010). Also, the role of resistance-harboring plasmids in dissemination of resistance was extensively studied (reviewed by Carattoli, 2009). In addition to the wealth of information that is currently still being discovered by applying these techniques on indicator micro-organisms, it has become clear that these methods can also be employed directly to biological samples. This enables analysis of resistance genes within the entire microbiota and investigates the so-called “resistome” thereby avoiding the potential bias that is introduced when selecting certain indicator micro-organisms.

## METAGENOMICS TO CHARACTERIZE THE AMR RESERVOIR

Metagenomics, refers to the study of metagenomes, genetic material recovered directly from environmental samples. It is the genomic analysis (analysis of all the DNA in an organism) applied to all the micro-organisms of a microbial ecosystem without previous identification (Lepage et al., 2013). Three different metagenomic approaches have been applied to study the resistome: targeted (PCR-based) metagenomics, functional metagenomics, and sequence-based metagenomics.

### TARGETED (PCR-BASED) METAGENOMICS

The accumulating evidence, showing that resistance genes are transferred within an ecosystem and across species, has highlighted the potential of application of easy-to-use PCR-based metagenomics. Numerous PCR methods are currently available for the detection of resistance genes or gene families. Furthermore, when real-time PCR is used to perform such analysis, (semi-)quantitative results can be generated and the relative abundance of resistance genes can be determined. This approach has been used to study the emergence of resistance over time in soil and water environments (Koike et al., 2007; Knapp et al., 2011). Knapp et al. (2010) showed that resistance to beta-lactamases, tetracyclines, and erythromycins in soil collected over a period of 70~years, significantly increased. A similar approach was applied to study the prevalence of resistance genes in oral biofilms (Kim et al., 2011) and very recently to study fluoroquinolone resistance in fecal samples of Vietnamese children (Vien le et al., 2012). The main drawback to using targeted PCR-based metagenomics is that the results are skewed towards known resistance genes and mechanisms. Importantly, evidence is accumulating of convergent evolution of resistance with different genes performing a similar function (Deschamps et al., 2009). Furthermore, sequence heterogeneity within a resistance gene found in different species may again skew the results towards mostly studied species. Nonetheless, the availability of this methodology within laboratories and possibilities for high-throughput analysis at limited costs make targeted (PCR-based) metagenomics a valuable tool in studying the resistome.**

### FUNCTIONAL METAGENOMICS

Functional metagenomics, involves the cloning of DNA fragments into a vector (e.g., a plasmid), and the subsequent expression into heterologous hosts (often *E. coli*). Next, these transformants are screened for the expression of resistance genes by growing them on antibiotic containing media at concentrations where the wild-type host strain is susceptible, where after antibiotic resistant clones are being sequenced (Schmieder and Edwards, 2012).

Sommer et al. (2009) characterized the resistance reservoir in saliva and fecal samples from two unrelated healthy individuals. The microbiota of these individuals was analyzed by functional screening of metagenomic DNA. Sequencing and annotation of clones conferring resistance to 13 different antibiotics enabled the discovery of 95 unique inserts containing functional antibiotic resistance genes. The majority of these genes were evolutionarily distant from known resistance genes, including 10 previously unidentified beta-lactamase gene families. This might reflect the existence of an unappreciated barrier between these bacteria with novel resistance genes and readily cultured human pathogens (Sommer et al., 2009, 2010; Schmieder and Edwards, 2012).

Recently a metagenomic library generated from the gut microbiota of four healthy humans was functionally screened for AMR genes against seven antibiotics (Cheng et al., 2012). This study confirmed the suitability of functional metagenomics to recover new AMR genes by identifying novel resistance genes against amoxicillin, D-cycloserine, and kanamycin.

Next, to the application of functional screening for resistance genes in the human microbiota, this approach has been used to detect the resistance reservoir in the gut microbiota of amongst others honeybees (Tian et al., 2012), chickens (Zhou et al., 2012), and gulls (Martiny et al., 2011).

One of the main limitations of functional screening is the dependence upon each gene’s ability to be expressed in surrogate hosts, typically *E. coli*. Resistance genes not being expressed by the surrogate host, e.g., because multiple genes are required that work together, regulatory elements are not being recognized, or posttranslational modifications are missing, leave these genes unidentified (false negatives). On the other hand the foreign gene may as well interact in novel ways with the cellular machinery of the surrogate host resulting in false positives (Schmieder and Edwards, 2012).

### SEQUENCE-BASED METAGENOMICS

In sequence-based metagenomics, DNA from an environmental sample is extracted, fragmented and size-separated, and randomly sequenced directly, without the need for culturing. The metagenomic sequences can subsequently be compared to international sequence databases to identify resistance genes (Schmieder and Edwards, 2012). The transition from Sanger sequencing to next-generation sequencing platforms (i.e., the Roche 454 sequencer, the Genome Analyzer of Illumina and the SOLiD system of Applied Biosystems) has resulted in a dramatic drop in costs and consequently have increased the number and size of metagenomic sequencing projects. These sequencing technologies yield lower contiguous read lengths and require greater genome coverage; however, their high-throughput reduces consumable costs and number of sequencing runs (Niedringhaus et al., 2011). Although the number of studies applying sequence-based metagenomics to characterize the human gut microbiome is rapidly growing, to our knowledge none of these studies specifically focussed on the AMR genes. Yet, the submission of these metagenomic libraries in public databases enables the *in silico* analysis of resistance elements within these studies.

Resistant bacteria from animals, zoonotic bacteria or intestinal commensals, can infect or reach the human population not only by direct contact, but also via food products of animal origin (van den Bogaard and Stobberingh, 2000) and through the application of animal feces as fertilizer in agriculture. As such, several studies have applied sequence-based metagenomics to characterize the virulence-associated and AMR genes in amongst others buffalo rumen (Singh et al., 2012), cattle feces (Durso et al., 2011), swine feces (Looft et al., 2012), and chicken cecum (Qu et al., 2008).

**FIGURE 1:**
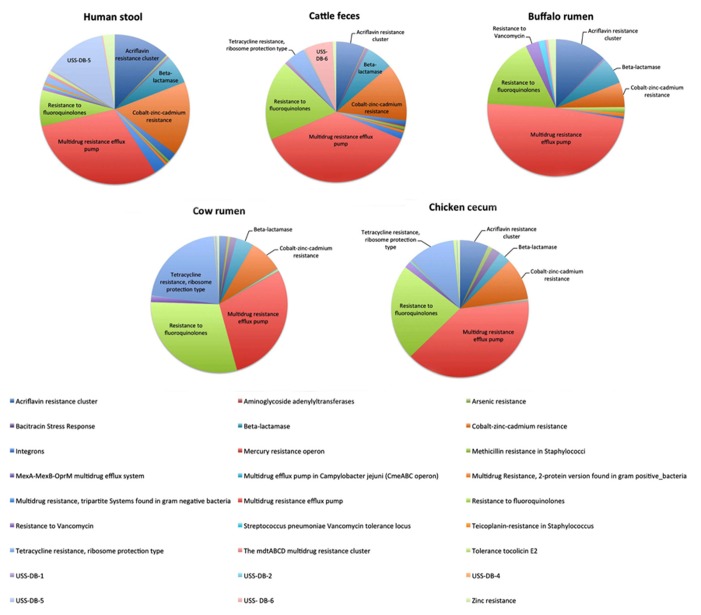
**Comparison of relative abundance of RATC genes in publicly available metagenomic datasets of human stool (MG-RAST #4444130.3), Cattle feces (MG-RAST #4448367.3), Buffalo Rumen (MG-RAST #4446901.3), Cow rumen (MG-RAST #4441679.3), and Chicken Cecum (MG-RAST #4440283.3)**. Data were derived from Singh et al. (2012). Sequences were annotated using the SEED database functional gene categories under the metagenome rapid annotation using subsystem technology (MG-RAST).

Over six percent (6.44%) of the sequences of the buffalo metagenomic library (Singh et al., 2012) and over eight percent (8.4%) of the sequences from the cattle fecal pool (Durso et al., 2011) could be mapped to virulence genes using the SEED database functional gene categories. In both datasets almost half of these sequences were associated with resistance to antibiotic and toxic compounds (RATC), the most frequently occurring functional group being multidrug resistance efflux pumps followed by fluoroquinolone resistance. The fluoroquinolone resistance genes are of particular interest because there is concern that the use of this class of antibiotics in veterinary medicine, particularly for food animals, may contribute to the development of resistance to this class of antibiotics in humans (Durso et al., 2011). Singh et al. (2012) compared their own buffalo rumen metagenomic dataset with datasets of amongst others cattle feces, cattle rumen, chicken cecum, and human stool (**Figure 1**). The multidrug resistance efflux pump and fluoroquinolone resistance were also the predominant RATC functional groups in the cattle rumen and chicken cecum libraries, whereas the fluoroquinolone resistance was less abundant in the human stool library.

As plasmids are important mediators of HGT of AMR genes, a more focussed sequencing of the plasmid metagenome (plasmidome/metamobilome) has also been applied (Walker, 2012).

Although with traditional metagenomic sequencing every single extra-chromosomal element within an environment can be sequenced (as long as the sequencing depth is sufficient), the proportion of chromosomal and extra-chromosomal genomes will always remain hugely in favor of chromosomes resulting in masses of redundant data when one is merely interested in the extra-chromosomal metagenome (Li et al., 2012) The research on the plasmidome is only in its infancy and has been applied to very few environments to date.

As described above, many resistance genes identified in studies applying functional metagenomics have low similarity to known genes. In sequence-based metagenomics, sequences with low similarity to known reference sequences cannot be easily identified, in other words this approach is generally limited to identifying genes that are already known (Schmieder and Edwards, 2012). The annotation power of sequence-based metagenomics will, however, keep improving, as long as functional metagenomic-based studies keep identifying novel AMR genes.

In addition, sequence-based metagenomics does not provide any information on the expression of the identified genes. On the other hand, sequence-based metagenomics provides a wealth of information not only on AMR genes, but on the entire gene content thereby enabling the identification of the community composition and metabolic profile. In particular these metagenomic data sets enable to look at which members of the bacterial community are carrying particular functional genes.

## CONCLUSION

Previous research on marker gut microbes has provided a wealth of information on amongst others AMS of these strains in different hosts, over time and at different geographic locations. In the current era of molecular techniques, there is still a place for culture-based studies as it is a necessity to assess antibiotic susceptibility. Yet, targeted, functional, or sequence-based metagenomics are required to gain more insight in the potential of the human gut microbioma as an AMR reservoir. These techniques have their own strengths and limitations and application of these techniques in population-based studies is only starting to emerge. Within the coming years, new metagenomics-based studies are likely to reveal many novel insights into this subject.

## Conflict of Interest Statement

The authors declare that the research was conducted in the absence of any commercial or financial relationships that could be construed as a potential conflict of interest.
